# Tensiomyographic Responses to Warm-Up Protocols in Collegiate Male Soccer Athletes

**DOI:** 10.3390/jfmk6040080

**Published:** 2021-09-23

**Authors:** Michael J. Redd, Tristan M. Starling-Smith, Chad H. Herring, Matt S. Stock, Adam J. Wells, Jeffrey R. Stout, David H. Fukuda

**Affiliations:** Institute of Exercise Physiology and Rehabilitation Science, School of Kinesiology and Physical Therapy, University of Central Florida, Orlando, FL 32816, USA; tstarling-smith@iwp-llc.com (T.M.S.-S.); chadherring@ucf.edu (C.H.H.); matt.stock@ucf.edu (M.S.S.); adam.wells@ucf.edu (A.J.W.); jeffrey.stout@ucf.edu (J.R.S.); David.Fukuda@ucf.edu (D.H.F.)

**Keywords:** tensiomyography, warm-ups, soccer, knee flexors, knee extensors

## Abstract

The mechanical properties of knee flexors and extensors in 15 collegiate male soccer players following different warm-up protocols [small-sided games (SSG), dynamic (DYN), and plyometric (PLY)] were evaluated. Tensiomyography (TMG) was used to assess contraction time (Tc), delay time (Td) and maximal displacement (Dm) of the rectus femoris (RF) and biceps femoris (BF) of both legs before and after each warm-up, while countermovement jump height variables, 20 m sprint, *t*-test and sit-and-reach were measured following the warm-ups. TMG was analyzed using a three-way [condition × time × leg] ANOVA, while performance variables were analyzed with a repeated measures ANOVA. Main effects of time were observed for BF-Tc (*p* = 0.035), RF-Td (*p* < 0.001), and BF-Td, (*p* = 0.008), and a main effect of condition was seen for RF-Tc (*p* = 0.038). Moreover, participants’ 20 m sprint improved following SSG (*p* = 0.021) compared to DYN and PLY. Sit-and-reach was greater following PLY (*p* = 0.021). No significant interactions were noted for the measured TMG variables. Warm-up-specific improvements were demonstrated in sprint speed and flexibility following SSG and PLY, respectively. The present study revealed changes in certain TMG measures following the warm-ups that suggest enhanced response of lower leg muscles regardless of specific activities used.

## 1. Introduction

Soccer is a sport comprised of intermittent high-intensity bouts of exercise, including maximal sprint efforts, jumping and multiple changes in direction [1,2]. The sport-specific demands of competitive soccer have been identified in a number of studies [3,4,5,6]. More specifically, high-level soccer players have been shown to commit 1000–1400 activity changes during a match [2,6,7,8] with varied high-intensity actions and multiple sprints at near-maximal intensities [8,9]. The preparation of soccer players during training sessions is critical for attaining optimal performance in match play, and the demands of a training session should closely mimic the competitive demands associated with games [10]. Sport coaches and strength coaches alike have sought to optimize player performance through the design and administration of a variety of training sessions. One of the key components to any training program or individual training session is the application of an appropriate warm-up designed to enhance performance [11,12,13].

Numerous studies have identified the benefits associated with participation in an organized warm-up protocol prior to athletic competition [12,13,14,15,16]. Studies have also shown improvements, such as increased preparedness and improvement in sport performance skills such as sprinting, jumping and change in direction, following training programs that incorporate plyometric exercises and jumps into their warm-up routines [1,12,15,17,18,19,20,21,22]. The previously identified benefits and improvements include physiological improvements, [1,15,17,18,19]. While there is some debate on which warm-up protocols produce the best results, there is a consensus that the warmup performed should consist of dynamic movements as well as an active stretching routine for agonist and antagonist muscles groups to reduce risk of injury and improve sport-related performance [12,13,14,15,23,24]. Other benefits of a dynamic warm-up include thermogenic effects, including: decreased viscous resistance of joints [12], increased blood flow to working muscles [25,26], increased anaerobic metabolism [14] and improved central nervous system functioning [25]. In support of this, active warm-up routines have been shown to improve performance in activities related to soccer such as sprinting, dribbling and striking a soccer ball [26].

Soccer teams use a variety of warm-up modalities at the start of training sessions such as a series of dynamic movements, reactive work with the ball during small-sided games or the incorporation of explosive plyometric movements into general routines [27]. Generally, these warm-ups have resulted in improved strength and power variables, such as sprint speed and jump height [1,17,18,23,26]. However, while contractile history has been shown to affect the performance of skeletal muscle [28], few studies have examined the contractile properties of muscles following a warm-up session [12,15].

Tensiomyography (TMG) is a non-invasive, indirect measure of superficial skeletal muscles’ contractile properties [29]. TMG measures are obtained by stimulating an individual muscle with a small electrical current and then recording a number of variables related to the mechanical properties of muscle contraction, such as: delay time (Td), contraction time (Tc), sustain time (Ts), relaxation time (Tr) and maximal displacement (Dm) [30]. TMG provides a valid and reliable means of immediate feedback [31], with the sensitivity to measure small changes in muscle contractile properties [32]. Subsequently, this method of evaluation has been used to detect neuromuscular status and muscle fatigue [33], which directly impacts muscle activity and performance. In a study by Loturco and colleagues [34], correlations were found between decreased Td, Tc and Dm, as measured by TMG, and greater scores on performance assessments. Pereira and colleagues [35] suggested that velocity of contraction, measured using TMG, may be a sensitive marker for assessing contractile property variations during performance assessments. Furthermore, TMG can be used to assess muscular fatigue by identifying parameters of muscle contraction such as Tc and Dm [36,37]. The aforementioned studies provide support that TMG variables may be affected by varying warm-up protocols.

Therefore, the purpose of this study was to compare the potential differences in the mechanical and physical characteristics of the knee flexors and extensors in collegiate male soccer players following different warm-up protocols. A greater understanding of the effects of various warm-ups on mechanical characteristics of the muscle may help to develop specific warm-up protocols that may lead to improved on-field performance. It is our hypothesis that TMG analysis will reveal that the warm-ups with a more explosive component will have the greatest impact on decreasing Tc and Dm of the muscles, and that the more continuous movement warm-ups may show increased signs of muscular fatigue.

## 2. Materials and Methods

### 2.1. Subjects

Fifteen male National Collegiate Athletics Association (NCAA) Division I soccer players (*n* = 15, 20.13 ± 1.26 years, 176.11 ± 7.85 cm, and 78.38 ± 7.50 kg) completed this study (Table 1). A convenience sample of 18 athletes from a single team initially consented; however, 2 athletes did not participate in any of the data collection sessions due to time conflicts. and 1 athlete was removed due to the identification of an ongoing injury that may have become exacerbated with continued participation.

All subjects were familiar with the warm-up protocols performed and the physical assessment tests used for this investigation. The protocols and testing were comprised of warm-ups and physical assessments typically performed by the men’s soccer team over the course of the competitive season. All athletes were free from injury and volunteered to participate in this investigation. In an attempt to eliminate the potential for fatigue-related performance decrements, subjects were asked to refrain from any workouts or training sessions during the duration of the protocol. The study was conducted according to the guidelines of the Declaration of Helsinki and approved by the University of Central Florida Institutional Review Board (IRB number SBE-17-13696; approval date: 31 January 2018). All components of the testing procedures were explained to each subject, and informed consent was received before the initial testing session. Neither the subjects nor the assessors were blinded for any of the testing sessions.

#### 2.1.1. Experimental Design

This study utilized a randomized crossover design to examine the effect of different warm-up protocols on performance and the physical and mechanical characteristics of knee flexors and extensors. The experimental design involved three different warm-up protocols: small-sided games (SSG), dynamic warm-up (DYN) or plyometric warm-up (PLY). The subjects were separated into three groups in order to accommodate weekly scheduling and limit the impact on training sessions. A random-sort procedure in Microsoft Excel was used by the study investigators to assign the order of protocols to each of the three groups. Each group completed the assigned protocol and associated testing at the same time of day for all sessions, while each session was followed by a 24 h off day before completing the next protocol. This 24 h period was intended to minimize fatigue and limit carry-over effects from the previous protocol sessions.

#### 2.1.2. Anthropometrics and Body Composition

Each subject’s height was measured with a portable stadiometer (Seca 213; Seca Corp., Chino, CA, USA) and body mass was measured with a calibrated standard medical scale (439 Physician Scale; Detecto, Webb City, MO, USA). Body composition was determined using a bioelectrical impedance analysis (BIA) device (Inbody 770, Biospace Co., Ltd., Seoul, Korea) that determined total fat and fat-free body mass by segmental multifrequency analysis, as recommended by the manufacturer. These anthropometrics and body composition measures were recorded as descriptive values on the subject’s first day of testing.

#### 2.1.3. Tensiomyography Measurements

The following procedures were used, as described by Jones and colleagues (2017), for assessment of the musculature of the leg utilizing TMG (TMG Measurement System, TMG-BMC Ltd., Ljubljana, Slovenia). For each muscle examined, the TMG sensor tip was placed at a location determined to measure maximal radial displacement of the selected muscle. All subjects were free from soft tissue or muscular injury at time of examination. Measurements were performed under static and relaxed conditions. Assessment of the RF was conducted with the subject in the supine position and the knee joint fixed at a 120-degree angle (with 180 degrees corresponding to full extension of the knee). The leg measured was positioned on a triangular wedge foam cushion to maintain a fixed knee angle. A digital displacement transducer, which incorporated a spring of 0.17 N·m^−1^, was set perpendicular to the muscle belly to acquire radial displacement of the selected muscle. The site for measurement of the radial displacement of the rectus femoris (RF) was determined by placing a transversal mark with a dermatological pen at 50% of the total length between the greater trochanter and the lateral condoyle of the distal end of the femur. The subject was then instructed to contract the quadriceps muscle of the leg being examined to palpate the rectus femoris and place a line longitudinally across the transversal line, creating an “x” landmark for sensor tip placement. Two square (5 × 5 cm) 2 mm-thick self-adhesive electrodes were placed symmetrically 5 cm (±5 cm) or 3 cm (±3 cm) distal and proximal to the sensor tip. This procedure was completed for both the right and left RF before the subject was repositioned in a prone position to examine posterior muscles of the leg. The site for measurement of the radial displacement of the biceps femoris (BF) was determined by measuring the distance between the ischial tuberosity and the lateral condoyle of the distal end of the femur and placing a transversal mark at 50% of the total length. The subject was then instructed to flex their knee against resistance, placed by the hand of the assessor at the ankle, to palpate the BF; a longitudinal line was drawn across the transversal line, creating an “x” landmark for sensor tip placement. This procedure was then repeated for both the right and left leg. Regarding electrical stimulation procedures, the pulse duration was 1 ms and the initial current amplitude was set at 50 mA. For each test, current amplitude was progressively increased by 10 mA increments until there was no difference in muscle displacement (Dm) or maximal stimulator output 100 mA was reached. For each subject, two consecutive measurements were recorded for each leg and muscle group, and averaged for analysis. Reliability of TMG, including the currently evaluated muscle groups, has been established in a review conducted by Martin-Rodriguez and colleagues [31], who reported high to excellent intraclass correlation coefficient (ICC) values for Dm (0.82–0.99), good to excellent ICC values for Tc (0.70–0.99) and low to excellent ICC values for Td (0.60–0.98). The same review reported standard error of measurement (SEM) values of ±1.0 mm for RF Dm, 1.20 ms for RF Td, 1.90 ms for RF Tc, 0.43 to 1.0 mm for BF Dm, 0.40 to 0.80 ms for BF Td and 1.06 to 5.60 ms for BF Tc.

### 2.2. Warm-Up Protocols

#### 2.2.1. Dynamic (DYN) Warm-Up

The DYN warm-up began with a 5-minute jog around the testing field. The next component was 9 min in duration and completed on the field at a distance of 12 yards, identified by 2 cones (cone A at start line and cone B placed at 12-yard point). The subjects completed a series of DYN patterns from cone A to B, then a jog from B to A with no rest between the DYN movement and jogging. Subjects were allowed a 5-second rest after the jog and before the next dynamic movement pattern began. This alternation between DYN pattern and jogging continued for the entire series of DYN patterns. The DYN warm-up is commonly utilized by the athletes and progressed from simple movements to a series of dynamic stretches and, finally, to more complex movements in multiple planes, including small skips, open the gate, close the gate, tall shuffle, lunge walk, side lunge walk, hamstring walk, knee hug, heel kicks, A-skip, B-skip, C-skip, low shuffle, carioca and lean–fall-sprint. The total activity time of the DYN warm-up was 15 min.

#### 2.2.2. Small-Sided Games (SSG) Warm-Up

The SSG warm-up began with a 5-minute jog around testing field followed by a 5-minute period involving a series of dynamic movement patterns over a distance of 12 yards from cone A to cone B, similar to that of the DYN but with no jogging involved. The final component of the SSG warm-up included players performing “rondo” drills that were common to the athletes, consisting of an individual player defending, or trying to win the ball from, a set of 4 offensive players positioned in a square approximately 7 m × 7 m in dimension. Players rotated positions in this drill, spending 1 min as a defender and 4 min as an offensive player, lasting a total of 5 min. The total activity time of the SSG warm-up was 15 min.

#### 2.2.3. Plyometric (PLY) Warm-UP

The PLY warm-up began with a 5-minute jog around the testing field. The next component was 4 min in duration and completed on the field at a distance of 12 yards, identified by 2 cones (configured similar to the other warm-up protocols.) This component of the PLY warm-up began with the following dynamic movements: small skips, open the gate, close the gate, tall shuffle, lunge walk, side lunge walk and hamstring walk. Unlike the DYN detailed above, these movements were completed from cone A to cone B and back to cone A, with no jogging between movement patterns; however, all subjects were required to observe a 5-second rest at cone A before beginning the next movement. For the final phase of the PLY warm-up, the subject completed a series of plyometric movements utilizing the same 12 yards distance from cane A to cone B and jog back to cone B. Subjects were allowed a 5-second rest after the jog and before the next PLY movement pattern began. The PLY component was commonly utilized by the athletes and included: double leg hops, single leg hops, lateral hops, double leg forward jump for distance, double leg explosive jump for height, single leg bounds, power skips and tuck jumps. The total activity time of the PLY warm-up was 15 min.

### 2.3. Performance Testing

#### 2.3.1. Jump Assessments

Countermovement jump (CMJ) tests were conducted using a validated optical timing system (MyJump V2.1), which recorded video on a tablet computer (iPad, Apple Inc., Cupertino, CA, USA) [38]. During the CMJ test, the subject was instructed to begin by standing with their hands at their waist, and then told to listen for an audible signal, which would alert the subject to bend his knees and maximally jump upward using arms for momentum. This CMJ was performed three times with 1 min of rest between each jump attempt. Variables analyzed during the CMJ included jump height (CMJ-h), flight time (CMJ-ft) and velocity (CMJ-v). Flight time was calculated for each jump after the take-off, and landing was identified in frames of the recorded video. The following equation: h = t^2^ × 1.22625 (h = jump height in meters, t = time in seconds) was used, as outlined by Bosco and colleagues [39], to determine jump height. Jump height from the application was found to have excellent reliability (ICC = 0.997, *p* < 0.001) and excellent agreement with countermovement jump height (CMJ-h), as measured using a force platform (ICC = 0.997, *p* < 0.001).

#### 2.3.2. Sprint Speed

Subjects were asked to perform 2 × 20 m maximal sprints on a natural grass surface with 1 min of rest between each sprint trial. Peak sprint speed was assessed by recording video of each sprint using a tablet computer (iPad, Apple Inc., Cupertino, CA, USA) and a mobile application (MySprint Apple Inc., Cupertino, CA, USA). The mobile application was specifically designed to use video analysis to determine the start time and finish time of each sprint using a frame-by-frame method. The tablet computer was mounted to a tripod and set at the 20 m point of the sprint, 18 m from the course, to ensure capturing the start and finish portions of the sprint. The sprint course was marked at 0, 5, 10, 15 and 20 m by vertical poles that were set to account for possible video parallax. That is, the poles were set not exactly at the specific distances (0, 5, 10, 15 and 20 m) but at the point where the subjects were viewed with the tablet computer to have crossed the maker with their hips and were at the target distance. Players were instructed to wear their normal soccer cleats for this test. The better of the 2 sprint trials time was recorded and used for further analyses.

#### 2.3.3. *t*-Test

The *t*-test course was set up with four cones—A, B, C and D. The subject began the *t*-test with both of his feet behind starting point A. The test started with the athlete being given a verbal “Go” command. On this command, the subject sprinted 10 yards forward to point B and touched the cone. Then, he shuffled 5 yards to the left and touched cone C. After that, he shuffled 10 yards to the right and touched cone D and then shuffled 5 yards to the left, back to point B. Then, the subject ran backward passing the finish line at point A. The time started on the player’s first movement from point A and stopped when he crossed the finish line. Time was measured using a hand-held stopwatch; this method was shown to have high ICC values (0.988) with electronic timing [40] and all *t*-test trials were timed by the same assessor. Each athlete performed this test two times and the faster time was recoded for further analysis.

#### 2.3.4. Sit-and-Reach

The subject was instructed to sit on the ground with knees straight, legs separated just enough to be comfortable, with the feet placed firmly against the sit-and-reach box. The arms were extended forward with the hands placed palms down on the upper surface of the box, which had a scale for distance printed on its horizontal surface (top side). In this position, the subject reached forward in a slow, consistent motion with no ballistic movement to the position of maximum reach. The test administrator stood close beside the scale and recorded the most distant line held and touched by the fingertips of both hands of the subject. If the hands reached unevenly, the hand reaching the shorter distance was used to determine the score. The score was recorded to the nearest half inch. If the reach appeared to be exactly half-way between two lines, the score was based on the last line actually touched.

#### 2.3.5. Statistical Analysis

All TMG measures data were analyzed using a three-way [condition (DYN vs. SSG vs. PLY) × time (pre-testing vs. post-testing) x leg (left vs. right)] repeated measures analysis of variance (ANOVA) with Holm post hoc analyses performed when appropriate. Due to technical issues during the TMG measurement process, only 14 full sets of data were available for the BF and 11 sets of data for the RF. All performance variable data (*n* = 15) were analyzed with a one-way repeated measures ANOVA with Holm post hoc analyses performed when appropriate. When violations of sphericity occurred, Greenhouse–Geisser corrections were utilized. Effect sizes were interpreted using Cohen’s d, in which d values of 0.8, 0.5 and 0.2 represented large, medium and small effect sizes, respectively [41]. Statistical software (JASP; Version 0.12; JASP Team, Amsterdam, The Netherlands) was used for data analysis. Results were considered significant at an alpha level of *p* ≤ 0.05. All data are reported as mean ± standard deviation.

## 3. Results

### 3.1. Tensiomyography

The TMG measurements performed pre- (PRE) and post-intervention (POST) are represented in Table 2.

No condition × leg × time interaction (F = 0.147, *p* = 0.755, η_p_^2^ = 0.014) or main effect of time (F = 3.953, *p* = 0.075, η_p_^2^ = 0.283) was noted for RF-Tc measures. However, a significant main effect of condition was observed (F = 3.887, *p* = 0.038, η_p_^2^ = 0.280). Follow-up analysis indicated that RF-Tc values were not significantly different between PLY and DYN (*p* = 0.087, d = 0.710 “medium”) or PLY and SSG (*p* = 0.068, d = 0.745 “medium”).

No condition × leg × time interaction (F = 0.710, *p* = 0.455, η_p_^2^ = 0.066) or main effect for condition (F = 4.309, *p* = 0.056, η_p_^2^ = 0.288) was noted for RF-Td. However, a significant main effect for time was observed (F = 29.890, *p* < 0.001, η_p_^2^ = 0.749). Post hoc analysis indicated that RF-Td decreased from PRE to POST (mean difference = 1.275 ms, 95% confidence interval = 0.755 to 1.795 ms).

No significant condition × leg × time interaction (F = 2.105, *p* = 0.162, η_p_^2^ = 0.139) or main effect for condition (F = 0.414, *p* = 0.552, η_p_^2^ = 0.031) was noted for BF-Tc. However, a significant main effect of time was observed (F = 5.537, *p* = 0.035, η_p_^2^ = 0.299). Post hoc analysis indicated that Tc of the BF muscle decreased from PRE to POST (mean difference = 1.890 ms, 95% confidence interval = 0.155 to 3.625 ms).

No condition × leg × time interaction (F = 0.306, *p* = 0.739, η_p_^2^ = 0.023) or main effect for condition (F = 0.537, *p* = 0.529, η_p_^2^ = 0.040) was noted for BF-Td. However, a significant main effect of time was observed (F = 9.749, *p* = 0.008, η_p_^2^ = 0.429). Post hoc analysis indicated that BF-Td decreased from PRE to POST (mean difference = 1.105 ms; 95% confidence interval = 0.341 to 1.870 ms).

No significant condition × leg × time interaction was noted for BF-Dm (F = 0.708, *p* = 0.502, η_p_^2^ = 0.052) or RF-Dm (F = 0.445, *p* = 0.541, η_p_^2^ = 0.043). Additionally, no main effects of time (F = 0.000, *p* = 0.984, η_p_^2^ = 0.000) or condition (F = 0.072, *p* = 0.931, η_p_^2^ = 0.007) were noted.

### 3.2. Performance Testing

The results for the *t*-test, sprint and sit-and-reach assessments are presented in Table 3. No significant difference was shown between conditions for *t*-test times (F = 0.943, *p* = 0.402, η_p_^2^= 0.063). A significant difference was found between conditions for 20 m sprint time (F = 4.719, *p* = 0.040, η_p_^2^= 0.252) with a reduction in sprint time following SSG compared to DYN (*p* = 0.021, d = 0.749 “medium”). A nonsignificant change between PLY and DYN for 20 m sprint time was noted (*p* = 0.083) with a “medium” effect size (d = 0.600). A significant difference was found between conditions for sit-and-reach scores (F= 4.394, *p* = 0.043, η_p_^2^= 0.239) with PLY, resulting in significantly greater scores than DYN (*p* = 0.021, d = 0.753 “medium”).

Results for the variables measured during the CMJ are presented in Table 2. Analysis of CMJ performance revealed no differences between conditions for CMJ-h (F = 0.372, *p* = 0.693, η_p_^2^ = 0.026), CMJ-ft (F = 0.406, *p* = 0.670, η_p_^2^ = 0.028) or CMJ-v (F = 0.430, *p* = 0.654, η_p_^2^ = 0.030).

## 4. Discussion

The primary findings of this study revealed that performing the warm-up protocols differentially affected performance assessments and the mechanical characteristics of the knee flexors and extensors in a sample of collegiate male soccer players. In terms of performance assessments, PLY resulted in significant improvements in sit-and-reach when compared to DYN and there was a significant reduction in 20-m sprint time following SSG compared to DYN. Time delay of both the RF and BF, and contraction time of the BF, decreased significantly from pre- to post-warm-up regardless of condition.

This study revealed a significant improvement in delay time, also termed reaction time [32], of the RF and BF from PRE to POST following each warm-up condition. This improvement, or decrease, in delay time following warm-up activities is most likely due to the post-activation potentiation effect generated by the exercises in the warm-up protocols. Previous studies have proposed that initiating neuromuscular potentiation may improve force production and performance [42]. Plyometric and jumping type exercises have been shown to be the most commonly prescribed methods to enhance post-activation potentiation [26]; however, the findings of this study may demonstrate similar acute responses following the exercises used in the three protocols. In support of this observation, improved post-activation potentiation has been postulated to increase the rate of force development in voluntary force production in dynamic muscle contractions [43].

Significant decreases in contraction time of the BF were noted from PRE to POST following each warm-up condition. This may be due in part to the improvements in the central nervous system nerve conduction rate associated with the increased muscle temperature following an active warm-up [25]. While the differences in Tc in the RF were not significant, medium effects were found for PLY compared to DYN (*p* = 0.087, d = 0.710 “medium”) and PLY compared to SSG (*p* = 0.068, d = 0.745 “medium”). While it was hypothesized that the plyometric type exercises would uniquely affect contraction time, the reason for this finding is unclear. We are unaware of any studies to date that have examined the effect of such training on contraction time using TMG; therefore, evaluation of plyometric training in this area of muscle mechanical response is warranted. It should also be noted that while the current data exceed previously reported SEM values for BF Tc from Simunic [44], they are below those reported by de Paula Simola [45] et al. and may need to be interpreted with caution.

No significant differences in maximal displacement were noted between warm-up conditions; however, previous studies have reported that this parameter is typically lower in soccer players compared to the general population. Low values of maximal displacement are usually indicative of higher muscle tone [46]. Therefore, based on the movement patterns and training of high-level soccer players, changes in this parameter may be difficult to elicit during a warm-up due to the relatively short duration of exercise.

Assessment of 20 m sprint time revealed a significant improvement following completion of SSG. While these findings are similar to a number of studies showing improvements in sprint time following active warm-ups and dynamic stretching routines [17,21,23,47], they contrast with Gabbett et al. [1], where no significant differences in sprint times were found for players completing an open-skill or closed-skill warm-up. This may be attributed to the specificity of the open-skill tasks examined in basketball players who performed warm-ups of similar distances and intensity regardless of ball possession [1], whereas the present study used a “rondo” drill in which the players intensity and distance were different than the comparison warm-ups. While not significant, a medium effect was noted for improved sprint times in PLY when compared to DYN. Although past studies have examined the effectiveness of dynamic warm-ups for improving sprint time, they have typically examined the difference in dynamic and passive warm-up routines [17,23]. We know of no other study to date that has compared the effect of a dynamic warm-up series vs. a warm-up inclusive of a large plyometric component on agility, sprint and jump performance. Rimmer and Sleivert [48] showed significant improvements in 40 m sprint time (following 8 weeks of sprint-specific plyometric training), while [49] showed improved 20 m sprint time after incorporating plyometric exercise the training programs of elite youth soccer players. The results associated with the latter improvements over short distances were attributed to the reduced ground contact time associated with plyometric exercises, which may have influenced the medium effect size for PLY compared DYN observed in the current study.

We also observed a significant improvement in sit-and-reach performance following PLY compared to DYN. The improved flexibility observed here may be attributed to the increased eccentric component associated with the movement patterns and muscle activation that accompanies repeated jumping type exercises [49]. Recurring bouts of eccentric training have been shown to shown to improve the muscle length–tension curve, which results in improvements in range of motion [50]. The present findings are consistent with previous studies that have shown improvements in flexibility associated with training that incorporates plyometric type exercises [22,49].

We observed no significant differences in measured CMJ variables between PLY, DYN and SSG warm-ups. This may be due to the acute nature of this investigation, in contrast to other studies that have used multiple training sessions incorporating plyometric exercises that have shown improvements in jump performance following warm-ups inclusive of jumping exercises [20,21]. Previous research has shown that skeletal muscle performance is affected by the contractile properties of muscle [28]. However, the single PLY session used in this study may not have increased contractile properties of the muscle associated with repeated activation of the stretch shortening cycle (SSC) induced by jumping-type exercises used in longer duration studies [42,51].

A potential limitation to this study is that the population examined was a homogenous group of male athletes with similar playing experience and competitive level from a single team. Future research should include groups of different playing experience or competitive level, or a control group of non-soccer athletes. Another possible limitation may be the acute nature of the warm-up protocol. Future research should examine whether there may be a difference in response if warm-ups are performed on a regular basis.

## 5. Conclusions

The warm-up protocols performed in this study represent variations of commonly used exercises for soccer athletes. The DYN warm-up examined herein positively impacted the contractile properties of leg muscles after performing a standardized series of dynamic movements. These exercises were familiar to the athletes and were performed in movement planes similar to those encountered in the competitive environment. Previous research has already established the performance improvements associated with DYN; however, they may have been less evident in the current study due to DYN being compared to SSG and PLY protocols rather than passive stretching or running-only warm-up interventions. The PLY exercises examined were shown to result in improved flexibility, as indicated by the improved sit-and-reach scores, and improved speed in the 20 m sprint, potentially as a result of post-activation potentiation.

The selection of warm-up protocols may be influenced by coaches and/or players attitudes or preferences for specific exercises. Another factor in warm-up selection and administration may be time constraints or the objectives of the coaching staff prior to match play, such as positioning, set piece formation, etc. While extensive research has been conducted on the benefits of warm-ups, there is no consensus on which combination of warm-up activities provide optimal results for sport performance. The disparity in previous findings may be due to the individual responses of athletes to the unique protocols or intensities at which they are performed, as well as differences in neuromuscular properties and fiber type composition. The findings of this study illustrate the benefits of incorporating more plyometric exercises and small-sided game situations to a standard pre-training or pre-competition warm-up. Future research should include studies examining the long-term effects of warm-up administration with larger samples of athletes at different levels of play and/or maturity status.

## Figures and Tables

**Table 1:** Means and standard deviations of selected characteristics by player position.

	GK (*n* = 2)	DF (*n* = 5)	MF (*n* = 5)	FW (*n* = 3)
Age (y)	20.33 ± 0.94	19.60 ± 1.02	20.4 ± 1.62	20.67 ± 0.47
Height (cm)	184.15 ± 3.81	174.24 ± 8.12	175.26 ± 4.25	175.26 ± 10.37
Body Mass (kg)	88.84 ± 5.06	77.82 ± 3.09	77.13 ± 6.12	74.42 ± 9.71
Body Fat (%)	16.15 ± 2.35	11.88 ± 2.01	12.34 ± 4.77	13.13 ± 1.13

GK = goalkeeper; DF = defender; MF = midfielder; FW = forward; *n* = sample size.

**Table 2:** Pre- and post-warm-up tensiomyography measurements (collapsed across right and left legs).

Muscle	Measure	Dynamic		Plyometric		Small-Sided Games	
		Pre	Post	Pre	Post	Pre	Post
Rectus Femoris (*n* = 11)	Tc (ms) ^#^	28.44 ± 4.41	27.01 ± 3.22	28.98 ± 4.60	28.75 ± 4.22	28.15 ± 3.68	27.19 ± 3.49
	Td (ms) *	24.89 ± 1.81	23.50 ± 2.04	25.15 ± 2.43	24.16 ± 1.98	24.46 ± 2.12	23.01 ± 1.39
	Dm (mm)	7.59 ± 1.95	7.21 ± 1.83	7.08 ± 1.97	7.87 ± 2.04	7.56 ± 2.58	7.16 ± 2.32
Biceps Femoris (*n* = 14)	Tc (ms) *	22.17 ± 3.60	20.71 ± 2.32	22.24 ± 3.65	20.37 ± 2.39	23.51 ± 4.32	21.17 ± 2.73
	Td (ms) *	21.93 ± 1.26	20.82 ± 0.95	21.78 ± 1.26	20.50 ± 1.24	21.90 ± 1.18	20.97 ± 0.99
	Dm (mm)	3.15 ± 0.80	3.06 ± 0.78	3.43 ± 1.11	3.19 ± 0.94	3.49 ± 0.78	3.16 ± 0.80

Tc = contraction time; Td = delay time; Dm = maximal displacement. * main effect for time (*p* < 0.05); ^#^ main effect for condition (*p* < 0.05).

**Table 3:** Performance test results (*n* = 15).

Test	Dynamic	Plyometric	Small-Sided Games
20 m Sprint (s)	2.75 ± 0.30	2.64 ± 0.13	2.62 ± 0.15 *
*t*-Test (s)	10.0 ± 0.27	9.99 ± 0.32	9.94 ± 0.30
Sit-and-Reach (cm)	34.17 ± 6.47	37.60 ± 6.98 ^†^	36.43 ± 7.29
CMJ Height (cm)	59.62 ± 8.10	59.31 ± 8.43	58.92 ± 8.93
CMJ Flight Time (ms)	695.80 ± 46.73	693.80 ± 49.13	691.40 ± 52.44
CMJ Velocity (m⋅s^−1^)	1.71 ± 0.12	1.70 ± 0.12	1.70 ± 0.13

* Significantly lower than plyometric (*p* < 0.05); ^†^ Significantly greater than dynamic (*p* < 0.05).

## Data Availability

The data presented in this study are available on request from the corresponding author. The data are not publicly available.
